# Stress Degradation Studies on Varenicline Tartrate and Development of a Validated Stability-Indicating HPLC Method

**DOI:** 10.3797/scipharm.1109-22

**Published:** 2011-12-05

**Authors:** Sudhakar S. Pujeri, Addagadde M. A. Khader, Jaldappagari Seetharamappa

**Affiliations:** 1 Department of Chemistry, Mangalore University, Mangalagangotri, India; 2 Department of Chemistry, Karnatak University, Dharwad, India

**Keywords:** Varenicline, Stability-indicating, Validation, Chromatographic assay, Chantix, Champix

## Abstract

A simple, rapid and stability-indicating reversed-phase liquid chromatographic method was developed for the assay of varenicline tartrate (VRT) in the presence of its degradation products generated from forced decomposition studies. The HPLC separation was achieved on a C18 Inertsil column (250 mm × 4.6 mm i.d. particle size is 5 μm) employing a mobile phase consisting of ammonium acetate buffer containing trifluoroacetic acid (0.02M; pH 4) and acetonitrile in gradient program mode with a flow rate of 1.0 mL min^−1^. The UV detector was operated at 237 nm while column temperature was maintained at 40 °C. The developed method was validated as per ICH guidelines with respect to specificity, linearity, precision, accuracy, robustness and limit of quantification. The method was found to be simple, specific, precise and accurate. Selectivity of the proposed method was validated by subjecting the stock solution of VRT to acidic, basic, photolysis, oxidative and thermal degradation. The calibration curve was found to be linear in the concentration range of 0.1–192 μg mL^−1^ (R^2^ = 0.9994). The peaks of degradation products did not interfere with that of pure VRT. The utility of the developed method was examined by analyzing the tablets containing VRT. The results of analysis were subjected to statistical analysis.

## Introduction

**Fig. 1:**
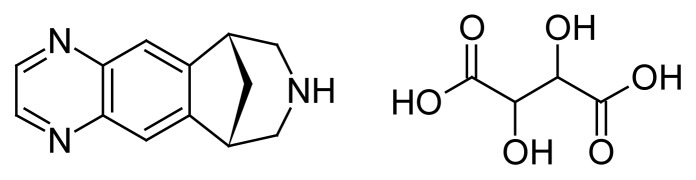
Structure of Varenicline tartrate (VRT)

Varenicline (VRT) chemically known as (6*R*,10*S*)-7,8,9,10-tetrahydro-6*H*-6,10-methano-pyrazino[2,3-*h*][3]benzazepine 2,3-dihydroxybutanedioate (1:1 salt, Fig. 1) was approved by US FDA as an aid to smoking cessation treatment [1–6]. It is a partial agonist and binds with high affinity and selectivity at α_4_β_2_ nAchR. It is used for the treatment of tobacco addiction [7–11]. Both nicotine and VRT bind to this receptor subtype. VRT blocks the ability of nicotine to activate α_4_β_2_ receptors and thus to stimulate the central nervous mesolimbic dopamine system, believed to be the neuronal mechanism underlying reinforcement and reward experience upon smoking [12].

Literature survey revealed a radiometric HPLC method for the analysis of VRT metabolism in *in vivo* and *in vitro* [13] and a stability-indicating HPLC method [14]. The reported method [14] has narrow linearity range (2–14 μg mL^−1^) and uses a high concentration of phosphate buffer (0.05 M) for elution. Further, it has poor system suitability parameters. In view of this, we have developed a sensitive HPLC method that overcomes the above limitations for quantitative determination of VRT in bulk sample and formulations. Further, stress studies on VRT were carried out. The added advantage of the proposed method is its specificity as it can be determined in the presence of its degradation products, excipients and additives.

## Experimental

### Chemicals and Reagents

Pure VRT was the kind gift from Pfizer Ltd, India. HPLC grade acetonitrile and trifluoroacetic acid was purchased from spectrochem, India. Ammonium acetate, hydrochloric acid, sodium hydroxide and hydrogen peroxide were obtained from Merck (Darmstadt, Germany). Water obtained from a Milli-Q water purification system (Millipore, MA, USA) was used throughout the study.

### Instrumentation

All HPLC measurements were made on a Waters 2695 separation module equipped with photo diode array detector 2996 module with data processing on Empower 2.0 version software. pH measurements were made on a pre-calibrated seven multi pH meter (Mettler Toledo Schwerzenbach, Switzerland). Mobile phase and sample/standard preparation were degassed using a sonicator (S. V. Scientific, India).

### Procedures

#### Preparation of Stock and Standard Solutions of VRT

A stock solution of VRT (1.0 mg/mL) was prepared in the diluent (water:acetonitrile, 1:1 v/v). Standard solutions of the drug were prepared by suitable dilution.

#### Analysis of Pharmaceutical Formulation

Ten tablets of VRT were finely powdered. An amount equivalent to 10 mg of the drug was weighed accurately and transferred into a 100 mL beaker. Using a mechanical stirrer, the powder was completely disintegrated in the mobile phase. The solution was filtered and the filtrate was made up to 10 mL with the mobile phase. It was further diluted for analysis.

### Validation of the Method

After method development, the validation of the current method was established as per the guidelines of ICH and USP.

#### Linearity and Range

**Fig. 2:**
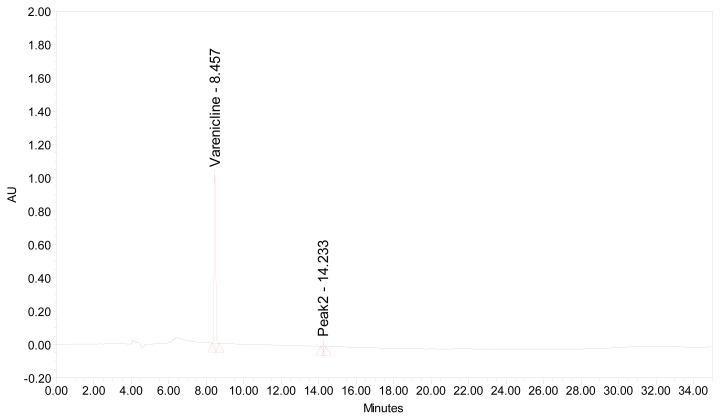
A typical chromatogram of VRT

To establish the linearity range, the standard solutions (10 μL) were injected in triplicate into the HPLC column and chromatograms were recorded. The peak area *versus* concentration data was treated by least-squares linear regression analysis. Typical chromatogram for VRT is shown in Fig. 2. The % RSD values were calculated.

#### Precision

Intra-day precision and inter-day precision were evaluated by analyzing six replicates of three different concentrations of the drug (50, 100 and 150 μg mL^−1^) on the same day and on different days, respectively. The respective %RSD values were calculated.

#### Accuracy

The accuracy of the assay method was evaluated by analyzing six replicates of three different concentrations of the drug (50, 100 and 150 μg mL^−1^). Further, it was also evaluated by fortifying a mixture of formulation sample with three known concentrations of the drug. The recovery of the added drug was determined.

#### Specificity and Selectivity

Specificity was established through the study of resolution factors of the drug peak from the nearest resolving peak and also among all other peaks. Further, the interference from excipients, impurities and degraded products on the assay of VRT was also examined.

#### LOD and LOQ

The LOD and LOQ were determined at a signal to noise ratio of 3:1 and 10:1, respectively, by injecting a series of test solutions of known concentrations within the linearity range. Precision study was also carried out at the LOQ level by injecting six pharmaceutical preparations.

#### Robustness

To determine the robustness of the developed method, experimental conditions were deliberately altered to check the reproducibility and quantitative recovery of the drug. This was carried out by maintaining the flow rate of the mobile phase in the range of 0.8–1.2 mL min^−1^ and column temperature in the range of 35–45 °C. Chromatograms were recorded and compared with those obtained under optimum chromatographic conditions, mentioned earlier.

#### Solution Stability and Mobile Phase Stability

The solution stability of VRT in the assay method was carried out by leaving both the test solutions of the sample and reference standard in tightly capped volumetric flasks separately, at room temperature, up to the study period of 48 h. The chromatograms of these solutions were recorded separately with an interval of 1 h up to 48 h and the peak responses were compared.

The mobile phase stability was carried out by assaying the freshly prepared bulk drug and formulation sample solutions against freshly prepared reference standard solution with an interval of 8 h. The same mobile phase was used throughout the experiment. %RSD values were calculated for mobile phase and solution stability experiments.

#### Forced degradation study

Forced degradation of the drug substance helps to establish the degradation pathways and the intrinsic stability of the drug. For this, the following experiments were carried out:

#### Oxidation of VRT

To study the oxidation of VRT, a 10.0 mL of stock solution of VRT (100 μg mL^−1^) was transferred to 20 mL amber colored volumetric flask and the volume was made up to 20 mL with 10% hydrogen peroxide. The flask was placed at 80 °C for 8 h, cooled to room temperature and the volume was readjusted with 10% hydrogen peroxide. Then the solution was filtered through a 0.45 μm syringe filter and 10 μL was injected into the liquid chromatographic system to detect the peak of degradant of the drug.

#### Thermal degradation of VRT

For this, 10.0 mL of VRT solution (100 μg mL^−1^) was transferred in to a 20 mL volumetric flask and diluted to the mark with mobile phase. The flask was closed and placed at 80 °C for 8 h, cooled to room temperature and volume of solution was readjusted with diluent. Chromatogram was recorded.

#### Degradation of VRT by Acid

For this, 10.0 mL of VRT solution (100 μg mL^−1^) was transferred into a 20 mL volumetric flask and the volume was made up to the mark with 1 M hydrochloric acid. The flask was placed at 80 °C for 8 h, cooled to room temperature and the volume was readjusted to 20 mL with 1 M hydrochloric acid. The solution was adjusted to neutral pH using 1 M sodium hydroxide and injected into the liquid chromatograph and chromatogram was recorded.

#### Degradation of VRT by Alkali

This was conducted by transferring 10.0 mL of VRT solution (100 μg mL^−1^) into a 20.0 mL volumetric flask and diluted to the mark with 1 M sodium hydroxide. The flask was kept at 80 °C for 8 h, cooled to room temperature and volume was readjusted to 20 mL with 1 M sodium hydroxide. The solution was neutralized with 1 M hydrochloric acid solution and then chromatogram was recorded.

### Photodegradation of VRT

For photodegradation studies, 10.0 mL of VRT solution (100 μg mL^−1^) was taken in to 20.0 mL volumetric flask and diluted to the mark with mobile phase. The flask was exposed to UV light for 8 h continuously. The experiment was also repeated with solid drug sample. The solutions of both were injected into liquid chromatograph separately and chromatograms were recorded.

## Results and Discussion

### Method Development and Optimization

The separation and run time of RP-HPLC depends on composition of the mobile phase or its solvent strength. Selection of the mobile phase is very critical for developing a method for the separation of impurities or degradants from the drug substance. The purpose of the buffer to be used in mobile phase is to control the pH. The selection of buffer is based on pKa of the compound. Maximum pH control of the mobile phase occurs at a pH that is equal to the pKa of buffer salt. Since the pKa of VRT is 9.2 at 25 °C, the selection of buffer with specific concentration and its pH becomes important. Basic buffer was found to be not suitable since unsymmetrical peaks were observed. Moreover, the peaks of degradants and pure drugs were not separated properly.

In the present study, symmetrical and resolved peaks were noticed with the mobile phase consisting of ammonium acetate buffer of pH 4.0 with an organic solvent, acetonitrile. The pKa of acetate salt (buffer) is 4.8 and it has a buffering range from 2.5 to 5.5, which is useful to develop a robust stability-indicating method.

Unwanted interaction between the drug substance and acidic silanol group on the surface of silica-based stationary phases is the major cause of poor column efficiency and selectivity. Fully reacted and end-capped column, Inertsil C18 was used to minimize the effect. Column compartment temperature was maintained at 40°C to improve resolution between the drug substance and degradation products.

To optimize the chromatographic conditions, the effect of composition of mobile phase, flow rate and the detection wavelength was investigated. Ideal chromatographic conditions were observed with the mobile phase consisting of ammonium acetate buffer containing trifluoroacetic acid (0.02 M; pH 4.0) and acetonitrile. Trifluoroacetic acid was used as a modifier to improve the peak symmetry and resolution. A flow rate of 1.0 mL min^−1^ was maintained throughout the analysis in gradient elution. Based on qualitative and quantitative results, we have selected the detector wavelength of 237 nm in the present study. Typical chromatogram is shown in Fig. 2.

#### Precision

The % RSD values for intra-day and inter-day assay precision were calculated and found to be less than 0.7% and 0.9%, respectively, indicating thereby that the method was sufficiently precise.

#### Accuracy

The % recovery values of VRT in pharmaceutical formulation ranged from 99.6 to 100.3. High percentage recovery values revealed that the proposed method is accurate and could be adopted for routine quality control.

#### Limit of Detection (LOD) and Limit of Quantification (LOQ)

The ICH guidelines were followed to calculate the values of LOD and LOQ and were observed to be 0.31 μg mL^−1^ and 0.97 μg mL^−1^, respectively. The %RSD value was noticed to be less than 1.0 % at LOQ concentration level.

#### Linearity

The calibration plot revealed the linearity in the range of 0.1–192 μg mL^−1^ (R^2^=0.9994).

#### Robustness

Under all the deliberately varied chromatographic conditions (flow rate and column temperature), the reproducibility of results was observed to be reasonably good. Hence, the proposed method has good robustness for the assay of VRT in bulk or in tablets.

#### Solution Stability and Mobile phase Stability

The % RSD values for the assay of VRT during solution stability and mobile phase stability experiments were found to be less than 0.7 %. This indicated that the sample solutions and mobile phases used during the assay were stable for at least 48 h.

### Forced Degradation Studies

**Fig. 3:**
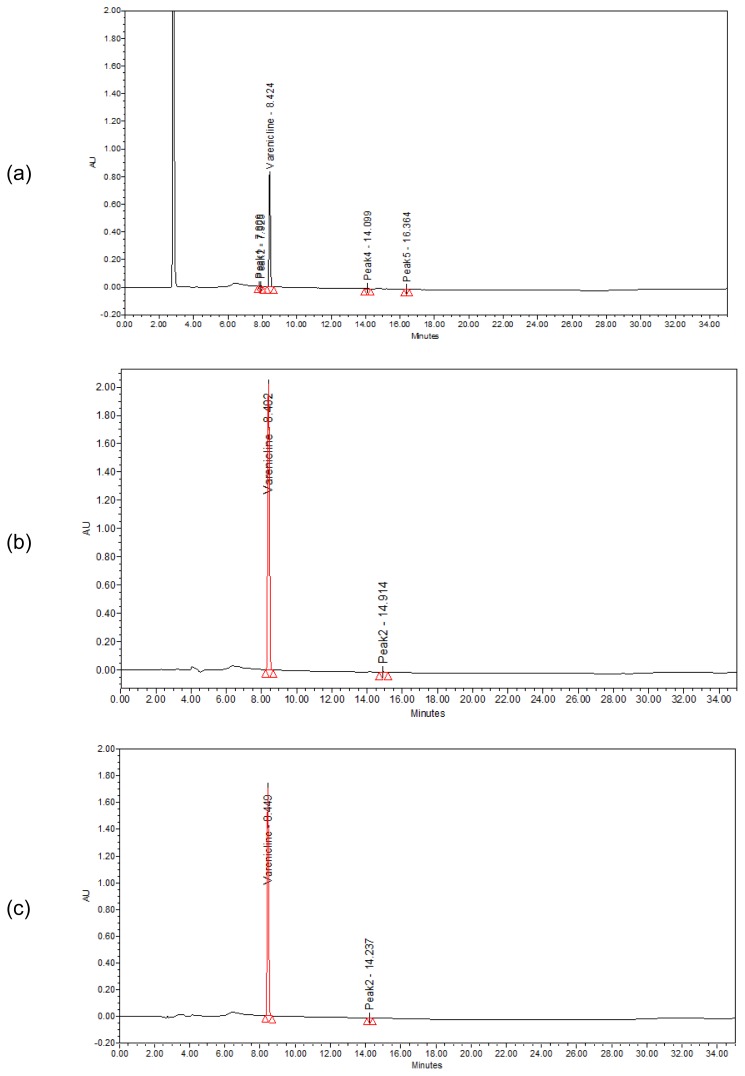

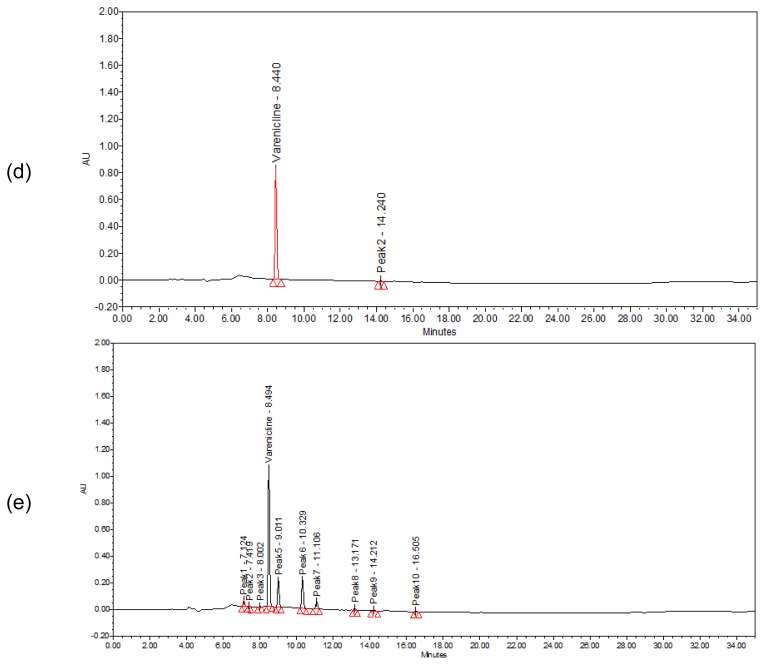
Typical chromatograms of VRT exposed to 10 % hydrogen peroxide (a), 80°C (b), 1 M hydrochloric acid (c), 1 M sodium hydroxide (d) and UV light (e)

Stress studies on VRT were carried out under oxidation, thermal stress, photolysis, acid and base hydrolysis conditions. It was found that the degradation was not observed in VRT sample when it was subjected to oxidation, thermal stress, acid and base hydrolysis (Figs. 3a, 3b, 3c and 3d). However, degradation products were found in photolysis stress conditions (Fig 3e).

**Fig. 4:**
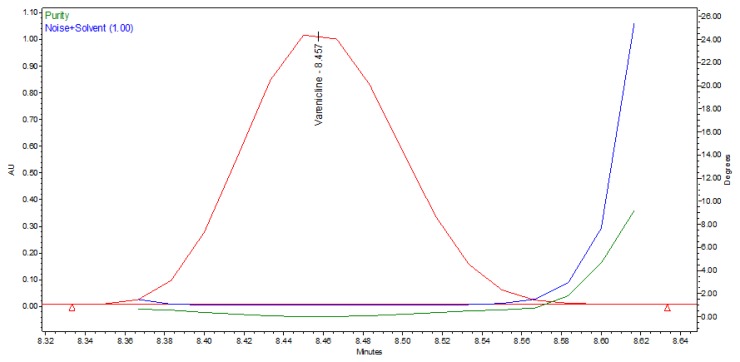
Purity plot of VRT

Peak purity test results confirmed that the VRT peak was homogenous and pure in all the analyzed stress samples. Lower purity angle value of VRT compared to that of the purity threshold (Table 2 and Fig. 4) revealed that the VRT was free from interference from its impurities and its degradants.

To avoid column damage at too acidic or too basic conditions generated during the stress studies and also to avoid peak splitting or unsymmetrical peak, the solutions were neutralized and chromatograms were recorded. Recovery results are shown in Table 3. Assay of the stressed samples was performed by comparison with reference standards and the mass balance (% assay + % degradation products) for stressed samples was calculated (Table 4). The developed HPLC method revealed that there was no interference from the impurities, degradation products and excipients to determine the assay of drug substance in pure and pharmaceutical formulation.

### Analysis of Pharmaceutical Preparations

The proposed method was successfully applied to the analysis of VRT in tablet and the results are shown in Table 5. The low values of %RSD indicated high precision of the method. High percent recovery values indicated that the commonly employed excipients microcrystalline cellulose, anhydrous dibasic calcium phosphate, croscarmellose sodium, colloidal silicon dioxide, magnesium stearate, opadry® white, opadry® blue and opadry® clear did not interfere in the analysis of VRT in tablets.

## Conclusions

The proposed method is observed to be precise, specific, accurate and stability-indicating. VRT can be determined in bulk powder, pharmaceutical formulation as well as in the presence of its degradation products by HPLC method. ICH guidelines were followed throughout the study for method validation and stress testing. In view of this, the proposed method could be adopted for quality control and routine analysis.

## Figures and Tables

**Tab. 1:** Gradient program

Time (min)	Mobile phase A (% v/v)	Mobile phase B (% v/v)	Elution
0.00–1.00	95	5	Isocratic
1.00–15.00	95→20	5→80	Linear gradient
15.00–25.00	20	80	Isocratic
25.00–30.00	20→95	80→5	Linear gradient
30.00–35.00	95	5	Re-equilibration

**Tab. 2:** System suitability parameters

Name	Retention time (min)	Purity angle	Purity threshold	USP tailing	USP Plate Count
VRT	8.417	0.161	1.016	1.070	48670

**Tab. 3:** Recovery results of VRT sample

Added (μg)^a^	Recovered (μg)	% Recovery	% RSD
50.0	49.8	99.6	0.5
100.0	100.1	100.1	0.7
150.0	150.4	100.3	1.2

a Average of six determinations.

**Tab. 4:** Summary of forced degradation results

Stress condition	Time (h)	% Assay of active Substance	% Mass balance (% assay + impurity)
Acid hydrolysis (1 M HCl) reflux at 80 °C	8	99.51	99.69
Base hydrolysis (1 M NaOH) reflux at 80 °C	8	99.48	99.67
Oxidation (10% H_2_O_2_) reflux at 80 °C	8	96.46	99.70
Thermal (80 °C)	12	99.60	99.68
Photolysis (254 nm)	24	63.28	99.67

**Tab. 5:** Analysis of VRT in pharmaceutical formulations.

Formulation	Labeled, mg	Found^a^, mg	% RSD	% Recovery
CHANTIX®	0.5	0.49	0.89	98.0
CHANTIX®	1.0	0.99	1.17	99.0

a Average of nine determinations.
